# Trends, prevalence and associated factors of obesity among adults in a rural community in Thailand: serial cross-sectional surveys, 2012 and 2018

**DOI:** 10.1186/s12889-020-09004-w

**Published:** 2020-06-03

**Authors:** Boonsub Sakboonyarat, Chanyut Pornpongsawad, Tanatip Sangkool, Chidapha Phanmanas, Nithitchaya Kesonphaet, Nirutti Tangthongtawi, Ammiga Limsakul, Ramita Assavapisitkul, Titipatara Thangthai, Patcha Janenopparkarnjana, Pijitra Varodomvitaya, Wichayada Dachoviboon, Janepoj Laohasara, Naphat Kruthakool, Sarawuth Limprasert, Mathirut Mungthin, Panadda Hatthachote, Ram Rangsin

**Affiliations:** 1 Department of Military and Community Medicine, Phramongkutklao College of Medicine, Bangkok, 10400 Thailand; 2 Phramongkutklao College of Medicine, Bangkok, 10400 Thailand; 3 Department of Internal Medicine, Phramongkutklao College of Medicine, Bangkok, 10400 Thailand; 4 Department of Pharmacology, Phramongkutklao College of Medicine, Bangkok, 10400 Thailand; 5 Department of Physiology, Phramongkutklao College of Medicine, Bangkok, 10400 Thailand

**Keywords:** Obesity, Trends, Rural community, Instant coffee-mix, Urine Na level

## Abstract

**Background:**

Obesity is one principle risk factor increasing the risk of noncommunicable diseases including diabetes, hypertension and atherosclerosis. In Thailand, a 2014 study reported obesity (BMI ≥25 kg/m^2^) in a Thai population aged ≥15 years was 37.5, 32.9 and 41.8% overall and among males and females, respectively. The study aimed to determine trends in the prevalence of obesity among adults residing in a Thai rural community between 2012 and 2018 and investigate the associations between obesity and behavioral factors.

**Methods:**

Serial cross-sectional studies were conducted in 2012 and 2018 among adults in Na-Ngam rural community. In 2012 and 2018, all 635 and 627 individuals, respectively, were interviewed using structured questionnaires related to demographics, risk behaviors, comorbidities and arthrometric measurement. Spot urine was collected by participants and obesity was defined as BMI ≥25 kg/m^2^. The risk factors for obesity were analyzed in the 2018 survey.

**Results:**

A total of 1262 adults in Na-Ngam rural community were included in the study. The prevalence of obesity was 33.9% in 2012 and 44.8% in 2018 (*P* < 0.001). The average BMI increased from 23.9 ± 4.2 kg/m^2^ in 2012 to 25.0 ± 4.52 kg/m^2^ in 2018 (*P* < 0.001). Obesity was associated with higher age (AOR 0.99; 95%CI 0.97–0.99), smoking (AOR 0.52; 95%CI 0.28–0.94), instant coffee-mix consumption > 1 cup/week (AOR 1.44; 95%CI 1.02–2.04), higher number of chronic diseases (≥1 disease AOR 1.82; 95%CI 1.01–2.68, > 2 diseases AOR 2.15; 95%CI 1.32–3.50), and higher spot urine sodium level (AOR 1.002; 95%CI 0.99–1.01).

**Conclusion:**

Our data emphasized that obesity constituted a serious problem among adults residing in a rural community. A trend in significant increase was found regarding the prevalence of obesity and average BMI in the rural community over 6 years. Effective public health interventions should be provided at the community level to reduce BMI. Moreover, modifiable risk factors for obesity should be attenuated to inhibit the progression of metabolic syndrome, noncommunicable diseases and their complications.

## Background

The prevalence of overweight status and obesity has been increasing both among males and females worldwide [1, 2]. Globally, in 2013, the estimated prevalence of adults with a body mass index (BMI) ≥25 kg/m^2^ totaled 36.9% among males and 38.0% among females [2]. In Thailand, the Thai National Health Examination Surveys V (NHES V) reported the prevalence of obesity (BMI ≥25 kg/m^2^) in a Thai population aged ≥15 years in 2014 was 37.5, 32.9 and 41.8% overall and among males and females, respectively [3].

Obesity is one principle risk factor leading to noncommunicable diseases including diabetes and hypertension [4, 5]. Additionally, obesity was confirmed to precipitate cardiovascular diseases, especially atherosclerotic cardiovascular diseases (ASCVD) [6, 7]. One recent report showed a low association between obesity and myocardial infarction, a moderate association with stroke and a high association with high blood pressure [8]. Thus, when the process of obesity is interrupted, these complications will be terminated. In addition, some dietary behaviors associated with obesity include salt intake and coffee consumption [9]. Related studies have reported an association between urine sodium levels as a proxy for salt intake, increasing BMI and metabolic syndrome [10, 11]. However, only limited information is available on factors potentially responsible for obesity among adults in a remote rural community, and the required information is essential to focus on preventing problems. Decreasing BMI will help to reduce cardiovascular risks and any complications. The present study was conducted directly in a rural population and reported trends in the prevalence of obesity among adults residing in a Thai rural community in 2012 and 2018. In addition, we investigated the associations of obesity and behavioral factors including urine sodium level.

## Methods

### Study design and subjects

This study was conducted in a rural community in central Thailand, 160 km from Bangkok: Na-Ngam rural community in Chachoengsao Province. The remote rural community houses approximately 1200 people. A serial cross-sectional study was conducted in September 2012 and December 2018. A total survey was used to collect information from the target population [12]. All 635 adults in 2012 and 627 adults in 2018 were interviewed. Inclusion criteria of the study comprised adults aged ≥20 years. People were excluded from the study when they did not reside in Na-Ngam community while the study was conducted.

This study was reviewed and approved by the Royal Thai Army Medical Department Institutional Review Board. Written informed consent was obtained from the participants with the WMA Declaration of Helsinki–Ethics principles for medical research involving human subjects.

### Data collection

The participants informed consent in Thai was obtained before participating in the study. The interviewers were well-trained before interviewing the participants. Face-to-face interviews were conducted using standardized questionnaires to obtain the information from the participants. One participant spent approximately 30 min to provide complete information.

### Measures

Standardized questionnaires, developed by the researchers for this study, covered information on demographics including sex, age, marital status, educational level, and occupation [see Additional file 1]. Self-reported information included diabetes mellitus, hypertension and dyslipidemia, exercise, smoking, alcohol consumption and instant coffee-mix consumption. A history of exercise was obtained using standardized questionnaires asking about the duration of daily or weekly physical activity, e.g., during the last 12 months, on how many days per week did you exercise? and how many minutes daily do you usually spend on one of those days?). Smoking was defined as those who currently smoked (within the last 12 months) and never smoked (patients who had never smoked, or who had smoked less than 100 cigarettes in their lifetime) [13]. Alcohol consumption was defined as those consuming within last 12 months and no alcohol consumption, i.e., those who never drank in their lifetime. Ex-smoker and ex-drinker were defined by smoke-free and alcohol-free for 12 months, respectively. Instant coffee-mix was defined as instant coffee mixed with milk and sugar ingredients. The number of cups consumed was calculated using questions about the number of cups daily, and weekly. Instant coffee-mix consumption behavior was computed as the median frequency of intake, in cups weekly. The number of cups weekly was categorized as: (1) ≤ 1 cup/week and (2) > 1 cup/week. A history of sugar-sweetened beverages consumption was obtained using standardized questionnaires asking about the number of cups daily, and weekly. Sugar-sweetened beverages consumption behavior was computed as the median frequency of intake, in cups weekly. The number of cups weekly was categorized as: (1) ≤ 1 cup/week and (2) > 1 cup/week. Body weight and height were measured using calibrated balance scales (to the nearest 0.1 kg) and stadiometer (to the nearest 0.1 cm), respectively (DETECTO, St. Webb City, MO, USA). BMI was calculated as body weight in kilograms divided by height in meters squared (kg)/(m^2^). BMI was classified in five groups, i.e., < 18.5 kg/m^2^, 18.5 to 22.9 kg/m^2^, 23.0 to 24.9 kg/m^2^, 25.0 to 29.9 kg/m^2^ and ≥ 30 kg/m^2^. Obesity was defined as BMI ≥25 kg/m^2^ [14]. The participants received a 60 ml. capacity screw cap container to store the collected urine, and participants were required to void the bladder. This first-pass urine was discarded. The urine passed thereafter was collected in the container provided approximately 40 ml. All urine samples were sent to a central laboratory within 6 h. Urinary sodium levels were measured using the VITROS 5,1 FS analyzer (Ortho Clinical Diagnostics, Neckargemünd, Germany). Urinary sodium was measured at the central laboratory for all urine samples of the participants.

### Statistical analysis

Data were analyzed using IBM SPSS Statistics for Windows, Version 23.0. Demographic data of participants were analyzed using descriptive statistics. Prevalence of obesity was calculated and presented as a percentage with 95% confident interval (95% CI). Student’s *t*-test was applied to compare continuous data while categorical data were compared with *chi*-square test. Generalized estimating equation (GEE) was used to analyze the changes in the BMI level and obesity for 97 individuals who were participated both in 2012 and 2018. Multivariable analysis was performed using logistic regression analysis to determine factors associated with obesity. The magnitude of association was reported as adjusted odds ratio (AOR) with 95% CI. Statistical significance was considered for *p*-value less than 0.05.

## Results

### Characteristics of the study participants

A total of 1262 adults in Na-Ngam rural community were included in the study, 635 participants in 2012, and 627 participants in 2018. In all, 97 subjects who participated in 2012 and 2018. The average age of participants was 48.9 ± 14.6 years and 54.9 ± 13.6 years in 2012 and 2018, respectively. Most participants graduated from primary school. Agriculture was the major occupation of the participants both in 2012 and 2018. Descriptive characteristics of the study participants by year are presented in Table 1.

**Table 1:** Demographic characteristics of participants

Characteristics	2012	2018
	***n*** = 635	***n*** = 627
	n (%)	n (%)
**Gender**		
Male	285 (44.9)	214 (34.1)
Female	350 (55.1)	413 (65.9)
**Age (years)** mean ± SD	48.9 ± 14.6	54.9 ± 13.6
20–29	54 (8.5)	29 (4.6)
30–39	130 (20.5)	51 (8.1)
40–49	172 (27.1)	130 (20.7)
50–59	118 (18.5)	178 (28.4)
60–69	99 (15.6)	148 (23.6)
≥ 70	62 (9.8)	91 (14.5)
**Marital Status**		
Married	496 (78.6)	486 (77.5)
Single	68 (10.8)	42 (6.7)
Widowed	43 (6.8)	69 (11.0)
Divorced	24 (3.8)	30 (4.8)
**Education**		
High school	75 (11.8)	89 (14.1)
Secondary school	65 (10.2)	50 (8.0)
Primary school	448 (70.6)	430 (68.6)
Less than primary school	47 (7.4)	58 (9.3)
**Occupation**		
Agriculture	362 (57)	279 (44.5)
Employment	110 (17.3)	112 (17.9)
Retail worker	74 (11.7)	76 (12.1)
Government officer	15 (2.4)	69 (11.0)
Non-occupation	74 (11.7)	91 (14.5)

*SD* Standard deviation

### Prevalence of obesity among adults in a rural community

**Fig. 1:**
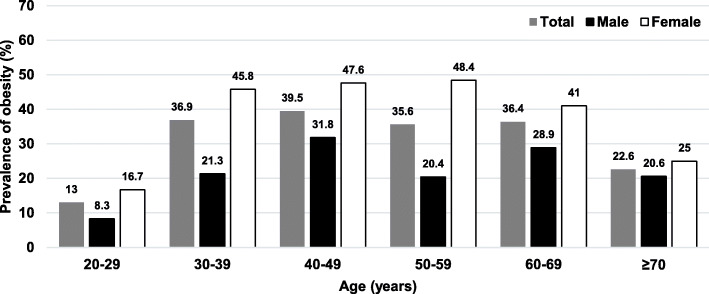
Prevalence of obesity among adults in a rural community in Thailand by age groups, 2012

**Fig. 2:**
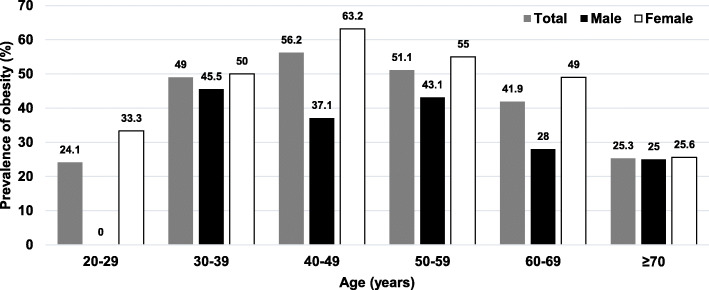
Prevalence of obesity among adults in a rural community in Thailand by age groups, 2018

In 2012, the overall average BMI among adults was 23.9 ± 4.2 kg/m^2^, while the overall prevalence of obesity was 33.7% (95%CI 30.2–37.6). The prevalence of obesity among females was 47.1%, while it totaled 24.2% among males. Figure 1 shows the prevalence of obesity among adults in 2012 stratified by age groups and sex. In 2018, the overall average BMI among adults was 25.0 ± 4.5 kg/m^2^, while the overall prevalence of obesity was 44.8% (95%CI 40.9–48.7). Among males, the prevalence of obesity was found at 32.7%, while the prevalence of obesity among females was 51.1%. Figure 2 illustrates the prevalence of obesity among adults in a rural community stratified by age groups and sex in 2018. The prevalence of obesity between participants with indoor occupation and outdoor occupation was equal, accounting for 45.9%.

### Trends in prevalence of obesity among adults in a rural community

**Fig. 3:**
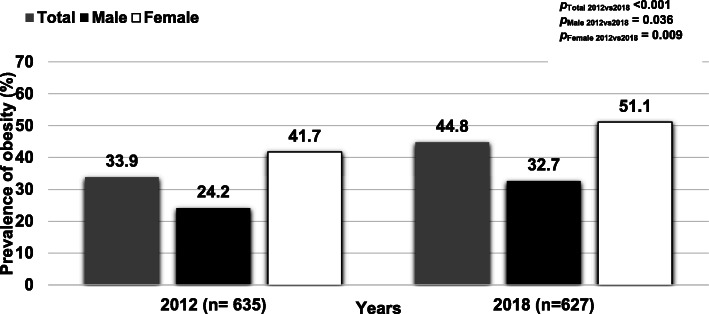
Trends in prevalence of obesity stratified by sex in a rural community in Thailand, 2012 and 2018

**Fig. 4:**
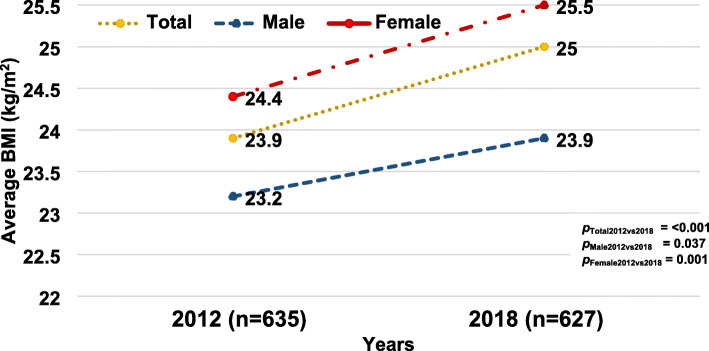
Average BMI among adults in a rural community in Thailand, 2012 and 2018

**Fig. 5:**
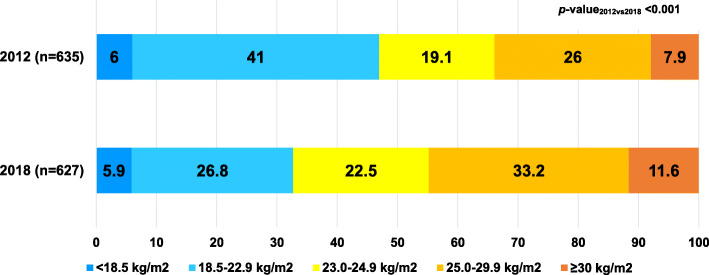
Proportion of BMI groups among adults in a rural community in Thailand, 2012 and 2018

Fig. 3 shows the trend in the prevalence of obesity stratified by sex in 2012 and 2018. The overall prevalence of obesity among adults increased significantly (*p* < 0.001); similarly, the prevalence of obesity among males and females significantly rose from 2012 to 2018. Figure 4 illustrates the average BMI in 2012 and 2018 revealing that BMI increased from 23.9 ± 4.2 kg/m^2^ in 2012 to 25.0 ± 4.5 kg/m^2^ in 2018 (*p* < 0.001). The average BMI increased both among males and females at 0.7 kg/m^2^ and 1.1 kg/m^2^ over 6 years, respectively. Figure 5 shows the proportion of BMI groups among adults comparing 2012 and 2018. In 2018, the proportion of BMI groups including 23.0 to 24.9 kg/m^2^, 25.0 to 29.0 kg/m^2^ and ≥ 30 kg/m^2^ increased in which the proportion of BMI ≥30 rose by 3.7% over 6 years. In all, 97 participants who enrolled in 2012 and 2018.

The repeated measurement analysis shows a significant increase in BMI level of the 97 individuals who participated both in 2012 and 2018 (*p* = 0.006).

### Associated factors of obesity among adults in a rural community in 2018

An additional table shows that univariate logistic regression analysis was performed to determine factors associated with obesity [see Additional file 2]. After adjusting for potential confounders, factors associated with obesity included smoking, instant coffee-mix consumption, spot urine sodium level and number of chronic diseases. Table 2 illustrates that the prevalence of obesity tended to be lower with older age adjusted odds ratio (AOR) 0.99; 95% CI 0.97–0.99). The prevalence of obesity among current smoker was lower than that of those who never smoked (AOR 0.52; 95% CI 0.28–0.94). Adults who consumed instant coffee-mix > 1 cup weekly tended to be at high risk for obesity compared with those who consumed ≤1 cup weekly (AOR 1.44; 95% CI 1.02–2.04). We found that increasing levels of spot urine sodium were not significantly associated with obesity among adults in a rural community (AOR 1.002; 95% CI 0.99–1.01). The increase in the number of chronic diseases of participants was associated with obesity as a dose response relationship (AOR 1.82; 95% CI 1.01–2.68 and AOR 2.15; 95% CI 1.32–3.50, respectively.

**Table 2:** Multivariable analysis factors associated with obesity among adults in a rural community in 2018 (*n* = 627)

Factors	BMI < 25 kg/m^**2**^	BMI ≥ 25 kg/m^**2**^	Adjusted Odds Ratio	95% CI	***p***-value
**Age (years)**	56.2 ± 14.9	53.4 ± 11.8	0.99	0.97–0.99	0.029
**Gender**					
Male	144 (67.3)	70 (32.7)	1		
Female	202 (48.9)	211 (51.1)	1.46	0.89–2.39	0.132
**Smoking**					
Never	224 (49.9)	225 (50.1)	1		
Ex-smoker	44 (62.0)	27 (38.0)	0.93	0.48–1.81	0.828
Current smoker	78 (72.9)	29 (27.1)	0.52	0.28–0.94	0.030
**Instant coffee-mixed drinking**					
≤ 1 cup/week	188 (57.1)	141 (42.9)	1		
> 1 cup/week	157 (52.9)	140 (47.1)	1.44	1.02–2.04	0.036
**Spot urine Na (mEq/L)**	122.3 ± 60.6	133.5 ± 74.1	1.002	0.99–1.01	0.116
^**c**^**Number of Chronic diseases**					
0	216 (61.7)	134 (38.3)	1		
1	81 (48.2)	87 (51.8)	1.82	1.01–2.68	0.018
≥ 2	49 (45.0)	60 (55.0)	2.15	1.32–3.50	0.002

Multivariable analysis (Enter); Age, Gender, smoking, coffee-mixed drinking, spot urine Na and number of chronic diseases

^c^Chronic diseases; diabetes mellitus, hypertension and dyslipidemia

## Discussion

This study constitutes a recent epidemiological study of obesity prevalence and associated factors for obesity among adults residing in a Thai rural community. These data provide essential evidence of the rising trend in the prevalence of obesity among adults residing in a rural community in 2012 and in 2018. Additionally, the study in 2018 illustrates the association between obesity and behavioral factors including comorbidities. Compared with the Thai NHES V in 2014 [3], the present study showed that obesity prevalence was higher, not only overall among adults, but higher than that in related reports among both males and females. Among females, the prevalence of obesity was significantly higher than that among males; comparatively, from a few related studies in Thailand [3, 15] and other countries, e.g., India [16], also indicating a high obesity prevalence among females. However, several studies in China [17, 18] and Lao PDR [19] reported that the prevalence of obesity among males was higher than that among females.

The prevalence of obesity among adults residing in a Thai rural community in 2012 and in 2018 significantly increased with an average BMI from 2012 to 2018. Additionally, we used repeated measurement among 97 individuals who participated in both 2012 and 2018. The results illustrated that BMI level significantly increased over 6 years. This finding was similar to the 2014 report indicating the global mean BMI rose among males and females [20]. Moreover, the average increase in BMI in the study was higher than that of the global increase among males and females accounting for 0.63 kg/m^2^ per decade and 0.58 kg/m^2^ per decade, respectively [20]. The phenomenon can be explained in that Thai society has transformed continuously to a more industrial society. Moreover, the number of rural areas has transformed continuously to urban regions resulting in economic development and decreased poverty levels in Thailand [21]. Thus, people can more easily access processed industrialized foods with a high in energy density foods leading to obesity [22, 23].

We found that, the prevalence of obesity increased with age, peaking at 50 to 59 years, and dropping at age more than 60 years. Similarly, related studies in China illustrated that the prevalence of obesity was higher among middle-aged people and decreased among older people [18]. Compared with other countries in Asia, the increase in BMI and prevalence of obesity in the present study was synchronous with the findings of other Asian countries, including Indonesia [24] and Korea [25]. Similarly, the recent study of Hatthachote et al. found a continuous increasing trend in prevalence of obesity among young Thai men over 8 years [26]. Obesity was confirmed as a potential risk factor for noncommunicable and ASCVD [4–7] When high BMI and obesity continue to progress, the prevalence of patients with noncommunicable diseases and their complications are more likely to increase. Thus, effective public health interventions should be implemented in the community to prohibit increased BMI.

The present study found that smoking behavior was associated with obesity. The participants who currently smoked were less likely to be obese when compared with adults who never smoked. The finding was consistent with related studies in Thailand that reported that smokers tended to have lower BMI than nonsmokers [27]. Similarly, recent large studies on the relationship between smoking and obesity conducted in the UK and Japan found that overall current smokers were less likely to be obese than those who had never smoked [28, 29]. This phenomenon can be explained by the effects of nicotine administration which involving the major appetite-suppressing component of tobacco; moreover, nicotine may act on the neural regulators of feeding [30]. Another explanation for the observation was that nicotine intake may stimulate the metabolic rate as the primary mechanism rather than diminish energy intake due to appetite suppression among smokers [31]. Although smoking can decrease obesity, it remains a serious health hazard for other issues including coronary heart disease and should be prevented for those reasons [32, 33].

We found that compared with participants consuming instant coffee-mix ≤1 cup weekly, participants who consumed > 1 cup weekly were more likely to be obese. Similarly, a recent study in Korea reported that high coffee consumption was positively associated with obesity as measured by BMI [9]. Additionally, the instant coffee-mix intakes were positively correlated with serum triglyceride level [34]. The finding can be explained by the high sugar level contained in the instant coffee-mix, directly increasing body weight and body fat accumulation [35]. Moreover, several reports have suggested that sugar-sweetened beverages were associated with the presence of dyslipidemia [36], type 2 diabetes mellitus [37] cardiovascular diseases [38] and metabolic syndrome [39, 40]. In the rural community, most residents are agriculturists who work hard daily with insufficient rest, so instant coffee-mix consumption is one alternative refreshment. Thus, lowering sugar level in coffee or black coffee consumption should be suggested. However, in a longitudinal study conducted in The Netherlands, coffee intake was unassociated with BMI [41]; additionally, in a cross-sectional survey of the National Health and Nutrition Examination Survey conducted in the US, coffee intake was unassociated with BMI and waist circumference among both males and females [42].

The present study reported that higher spot urine sodium levels held a positive relationship to obesity, however this was not statistically significant. A few related studies conducted in the UK, Korea and Brazil supported this finding in that having high sodium excretion levels showed increased odds of overweight status and central obesity [10, 43, 44]. In addition, a population-based cohort study conducted in the US reported that an increase in urinary sodium-to-potassium ratio related to higher total body percentage fat [45]. The phenomenon could be explained along with salt intake measured by urine sodium [44]. One related study showed that salt intake was related to obesity through energy intake such as the co-existence of high salt and high energy junk food diets [46]. The mechanism for a direct effect remains unclear. A possible mechanism is that consuming high levels of salt increases the volume of extracellular water, resulting in increased weight [47]. Another mechanism is that salt could directly increase adipose tissue and body fat [48]. Similarly, one epidemiological study conducted among adolescents revealed a positive relationship between salt consumption and subcutaneous abdominal adipose tissue, as well as BMI [49].

Participants with a higher number of chronic diseases including type 2 diabetes, hypertension and dyslipidemia were more associated with obesity. The finding was consistent with one related report in Ireland reporting that a large proportion of chronic disease was attributable to increased BMI [50]; additionally, one study across low to middle income countries involving 31,118 participants found that associations of obesity with diabetes and hypertension were strong [51]. Obesity has been confirmed to increase the risk of metabolic syndrome [52, 53] and noncommunicable diseases resulting in ASCVD [4, 5, 7]. Moreover, the study conducted in Ireland showed that a relatively modest reduction in average BMI had the potential to create a significant impact on the burden of chronic disease [50]. Thus, reducing BMI should be recommended to those with obesity to alleviate any noncommunicable diseases.

The study employed two cross-sectional surveys in 2012 and 2018, making it difficult to establish a cause-and-effect relationship between associated factors and obesity. Because the study employed a serial cross-sectional design, some limitations were encountered regarding the behavioral factors and spot urine Na level that were not included in the 2012 survey. Therefore, we could not combine the two surveys in the same data analysis for risk factors. However, the 2012 survey could provide demographic characteristics and the baseline prevalence of obesity. Thus, only the second survey in 2018 was used to obtain the associated risk factors of obesity in our current study. Some variables were collected very broadly as the number and type of alcohol drinks together with the number of cigarettes consumed and smoking frequency were not recorded; however, the associations between factors and outcomes were able to be presented. Finally, social desirability bias might also have existed in the study due to face-to-face interviews. However, the interviewers were well-trained and used standardized surveys.

Our findings suggested that modifiable risk factors for obesity should be improved. Adults with obesity especially those residing in a rural community should be targeted for more educational interventions in raising awareness regarding reducing their high BMI levels and associated complications and adjusting their daily activities. Our study may not be generalized to the whole country but may reflect challenges of individuals residing in rural communities of Thailand.

## Conclusion

Our data emphasized that obesity constituted a serious problem among adults residing in a rural community. A significant increasing trend in the prevalence of obesity was observed and average BMI in the rural community was found to increase over 6 years. Effective public health interventions, especially modifying dietary behavior, should be provided in the community to reduce BMI. The modifiable risks factors for obesity should be attenuated to inhibit the progression of metabolic syndrome, noncommunicable diseases and their associated complications.

## Supplementary information

**Additional file 1.** Standardized questionnaires for the study (English version).

**Additional file 2. **An additional table, univariate analysis factors associated with obesity among adults in a rural community in 2018 (*n* = 627).

## Data Availability

The dataset analyzed is available from the corresponding author on reasonable request.

## Abbreviations

BMI: Body mass index
CI: Confident interval
AOR: Adjusted odds ratio
SD: Standard deviation
HT: Hypertension
DM: Diabetes mellitus
DLP: Dyslipidemia
ASCVD: Atherosclerotic cardiovascular diseases
GEE: Generalized estimating equation
