# Supplementary material for: Trends, prevalence and associated factors of obesity among adults in a rural community in Thailand: serial cross-sectional surveys, 2012 and 2018
Source: BMC Public Health. 2020 Jun 3;20:850. doi: 10.1186/s12889-020-09004-w (PMC7271557; doi:10.1186/s12889-020-09004-w)
Supplement: Supplementary file 1 — Additional file 1. Standardized questionnaires for the study (English version). [file 12889_2020_9004_MOESM1_ESM.docx]

| **Case Record Form**  **Prevalence and associated factors of obesity among adults in a rural community in Thailand** | |
| --- | --- |
|  | |
| **Date** (Ex 01 /12 /2018) □□/□□/□□□□ | |
| 1. Sex ❑ 1. Male ❑ 2. Female | |
| 2. Birth year □□□□ (Ex. 1991)  **Age** □□ **years** (Ex. 27 years) | |
| 3. Body weight □□□.□ kg. | 6. Height □□□.□ cm. |
| 4. Occupations  ❑ 1. Agriculturists ❑ 2. Employment ❑ 3. Retail workers   ❑ 4. Government officers ❑ 5. Non-occupation ❑ 6. Others…………………………………… | |
| 5. Educational level  ❑ 1. Less than primary school ❑ 2. Primary school   ❑ 3. Secondary school ❑ 4. High school  ❑ 5. Bachelor ❑ 6. Others…………………………………….. | |
| 6. Marital status  ❑1. Single ❑ 2. Married ❑ 3. Widowed ❑ 4. Divorced | |
| 7. Comorbidities (Choose more than 1)  ❑ 1. No ❑ 2. Diabetes Mellitus  ❑ 3. Hypertension ❑ 4.Dyslipidemia  ❑ 5. Others………………………………. | |
| 8. During last 12 months, did you regularly smoke?  . ❑ 1. Current smoked  ❑ 2. Never (patients who had never smoked, or who had smoked less than 100 cigarettes in their lifetime).  ❑3. Ex-smoker (smoke-free for 12 months) | |
| 9. During last 12 months, did you regularly drink any alcohol?  ❑ 1. Current drinker (consuming within last 12 months)  ❑ 2. Never ( never drank in their lifetime.)  ❑ 3. Ex-drinker ( alcohol-free for 12 months | |
| 10. During the last 12 months, how many days per week did you exercise?  □ Days/week  11 How many minutes daily do you usually spend on one of those days?  □□□Minutes/day | |
| 12. How many days per week did you consume sugar-sweetened beverages?  □ Days/week  13.How many cups of sugar-sweetened beverages consumption per day?  □□Cups/day | |
| 14. How many days per week did you consume Instant coffee-mix ( instant coffee mixed with milk and sugar ingredients) ?  □ Days/week  15. How many cups of instant coffee-mix consumption per day?  □□Cups/day | |
| 16.Spot urine sodium =□□□ mmol/L | |

**-----END----**
